# Highest Resolution In Vivo Human Brain MRI Using Prospective Motion Correction

**DOI:** 10.1371/journal.pone.0133921

**Published:** 2015-07-30

**Authors:** Daniel Stucht, K. Appu Danishad, Peter Schulze, Frank Godenschweger, Maxim Zaitsev, Oliver Speck

**Affiliations:** 1 Biomedical Magnetic Resonance, Otto-von-Guericke University, Magdeburg, Germany; 2 Institute of Biometry and Medical Informatics, Otto-von-Guericke University, Magdeburg, Germany; 3 Department of Radiology, University Medical Center Freiburg, Freiburg, Germany; 4 Center for Behavioral Brain Sciences, Magdeburg, Germany; 5 Leibniz Institute for Neurobiology, Magdeburg, Germany; 6 German Center for Neurodegenerative Disease (DZNE), site Magdeburg, Germany; University of Ulm, GERMANY

## Abstract

High field MRI systems, such as 7 Tesla (T) scanners, can deliver higher signal to noise ratio (SNR) than lower field scanners and thus allow for the acquisition of data with higher spatial resolution, which is often demanded by users in the fields of clinical and neuroscientific imaging. However, high resolution scans may require long acquisition times, which in turn increase the discomfort for the subject and the risk of subject motion. Even with a cooperative and trained subject, involuntary motion due to heartbeat, swallowing, respiration and changes in muscle tone can cause image artifacts that reduce the effective resolution. In addition, scanning with higher resolution leads to increased sensitivity to even very small movements. Prospective motion correction (PMC) at 3T and 7T has proven to increase image quality in case of subject motion. Although the application of prospective motion correction is becoming more popular, previous articles focused on proof of concept studies and technical descriptions, whereas this paper briefly describes the technical aspects of the optical tracking system, marker fixation and cross calibration and focuses on the application of PMC to very high resolution imaging without intentional motion. In this study we acquired in vivo MR images at 7T using prospective motion correction during long acquisitions. As a result, we present images among the highest, if not the highest resolution of in vivo human brain MRI ever acquired.

## Introduction

### High resolution MRI

Ultra High Field (UHF) MRI has become increasingly available for fields such as biology, neuroscience or diagnostic imaging. Its benefits are an increased SNR and the potentially higher resolution showing a high level of anatomical detail. As MRI is non-invasive, the new developments for high-resolution imaging could be used for non-invasive in vivo brain MRI histology, impossible to obtain with traditional histology.

High resolution imaging of the living human body faces many challenges: resolution, scan time, and SNR describe the limiting factors of an MRI scan. Usually, a compromise in this triangle has to be found, often in favor of one of its corners. Aiming for high resolution and good SNR both increase scan time: The SNR of the MR data and of the resulting image is proportional to the voxel volume. Hence, SNR is decreasing for higher resolutions. In addition more voxels are needed to cover the same volume of interest (VoI) which increases the encoding time. If the SNR is to be kept at the same value, multiple averages will become necessary, which easily brings the scan time into the range of several hours and raises new problems as discussed in the next section.

### High resolution Imaging on UHF whole body systems and the need for motion correction

Improvements in MR hardware such as more efficient RF-coils with higher numbers of receive channels and stronger or faster gradients can help to increase SNR. Commonly it is assumed, that in sample-noise dominated acquisitions SNR scales linearly with magnetic field strength. According to [1], however, SNR scales more than linearly, hence the use of ultra-high field systems is an increasingly popular approach to gain SNR and thus to allow for very high resolution imaging.

Studies have shown that a higher resolution can have a strong impact, e.g. when measuring the cortical thickness from MRI scans [2]. The possibility to improve the spatial resolution at UHF for high resolution human brain imaging has already been shown in post mortem studies on a 7 T whole body scanner [3, 4] with an isotropic resolution of 140 μm and full coverage of a formalin-fixed human brain. Many publications in the field of high resolution in vivo imaging of the human brain describe imaging at ultra high field strengths of 7T or above but otherwise conventional hardware. Especially the possibility to visualize alterations caused by aging or neurodegenerative diseases at a high level of detail has evoked attention. Higher detail would allow for the identification of smaller pathological changes at an earlier stage. High resolution 7T gradient echo data of microvascular abnormalities, e.g. in multiple sclerosis lesions [5], in gliomas [6] or changes in the dentate granule cell layer of schizophrenia patients [7] were presented with resolutions between 0.196 × 0.196 × 2.0 mm and 0.232 × 0.232 × 1.5 mm. 7T time-of-flight (ToF) data of healthy subjects and patients with aneurysms or arterial-venal malformations were presented in [8–10] with resolutions between 0.22 × 0.22 × 0.41 mm and 0.43 × 0.43 × 1.2 mm. These studies also show the superior quality of UHF ToF data compared to 3T. In an ultra-high resolution 7T MRI data repository [11], Forstmann et al. make MP-RAGE data of 0.6mm and 0.7mm isotropic resolution available to the public. Lenglet et al. presented 7T MP-RAGE images of the basal ganglia with resolutions of 0.4 × 0.4 × 1.0 mm. Keuken et al. [12] report that in their high resolution study using 0.7 mm isotropic MP-RAGE data and 0.5 × 0.5 × 0.6 mm gradient echo data, the data set of one subject could not be used because of severe motion artifacts. To the best of our knowledge, the highest resolution in vivo images from the human brain before our current study are acquisition-weighted data presented in [13]. They were acquired at 9.4T with a resolution of 0.13 × 0.13 × 0.8 mm and no motion correction was applied. Even though no distinct motion artifacts are visible, the authors assume that applying some motion correction would further improve image quality.

For in vivo imaging, a substantial limiting factor for very high resolution imaging is set by the subjects’ ability to stay motionless in the same position and orientation during the entire examination. Conventional MR-imaging is carried out under the assumption that the patient does not move during the scan. Patient motion results in image artifacts [14] and thus limits the effective resolution and influences the results of analyses and calculations, such as segmentation [15] or gray matter volume and thickness estimates [16]. During the scan time, subject motion has to be below a threshold determined by the image resolution and the motion pattern [17]. Restraints such as cushions and pads can help to decrease motion, but cannot entirely prevent it and are often uncomfortable for longer scans. Nevertheless, even for shorter scan times in clinical routine at 1.5 T and 3 T, artifacts such as ringing, ghosting and blurring caused by data inconsistencies due to motion during the image acquisition are well-known problems.

The main sources of subject motion are muscle relaxation (leading to “long term motion” i.e. a slow drift of several millimeters during long acquisitions(minutes), heartbeat and breathing (leading to periodic motion below 1 millimeter) and single motion events such as coughing, sneezing and swallowing (often leading to brief large-scale motion). Depending on the quality and the periodic nature of the motion, the intensity, frequency and the temporal occurrence during the scan (which k-space lines are affected), these motions cause artifacts such as blurring and ghosting of different strength along the phase-encoding direction of the image [14, 18, 19].

Longer scan times for very high resolution imaging at ultra-high field increase the incidence of motion artifacts, even with cooperative subjects, which presents a new “biological resolution limit”. This barrier originates from involuntary subject motion, i.e. breathing, heartbeat or muscle relaxation leading to motion artifacts as was shown by Herbst and colleagues [20]. Such artifacts diminish the high resolution capabilities of ultra-high-field systems for imaging of human subjects. Therefore effective motion correction techniques need to be applied for very high resolution imaging.

### Motion Correction techniques

Aside from motion correction techniques such as holding the breath for short acquisition times or ECG synchronization [21, 22], more sophisticated procedures such as the use of navigators [23–27], optimized encoding schemes and self navigated sequences (e.g. PROPELLER [28]) can be used. Information about the subject pose can be used to correct for motion either in real time (prospectively) or offline after the data has been collected (retrospectively). Spin history effects caused by through-plane motion are not corrected by retrospective correction. As the scan itself is not adjusted, the motion may cause parts of the volume of interest to move out of the field of view.

In contrast to retrospective techniques, PMC ensures that the k-space sampling density stays approximately homogeneous. The motion data can be obtained in various ways, e.g., by navigator techniques ([29, 30]), by MR imaging and registration algorithms, by using micro RF-coils [31, 32] or by an external tracking device. A variety of optical tracking systems exists. Stereoscopic tracking systems, e.g. infrared based tracking, often track retro reflective markers [33]. Single camera systems can use retrograde markers [34], or track object features such as facial structures or 2D patterns [35] to calculate pose information. Most techniques assume a rigid body transformation with 6 degrees of freedom (DoF), namely three rotations and three translations along the MRI coordinate system. If an external tracking system is used, no extra imaging time is required to obtain head pose information. A detailed review of PMC in human brain imaging is given by Maclaren et al. [36].

This work evaluates the applicability and efficacy of a PMC system consisting of a single camera and a moiré phase tracking (MPT) marker [37, 38] for very high resolution in vivo human brain MRI at 7 T to overcome the “biological resolution limit” described above.

## Materials and Methods

### Tracking system

**Fig 1:**
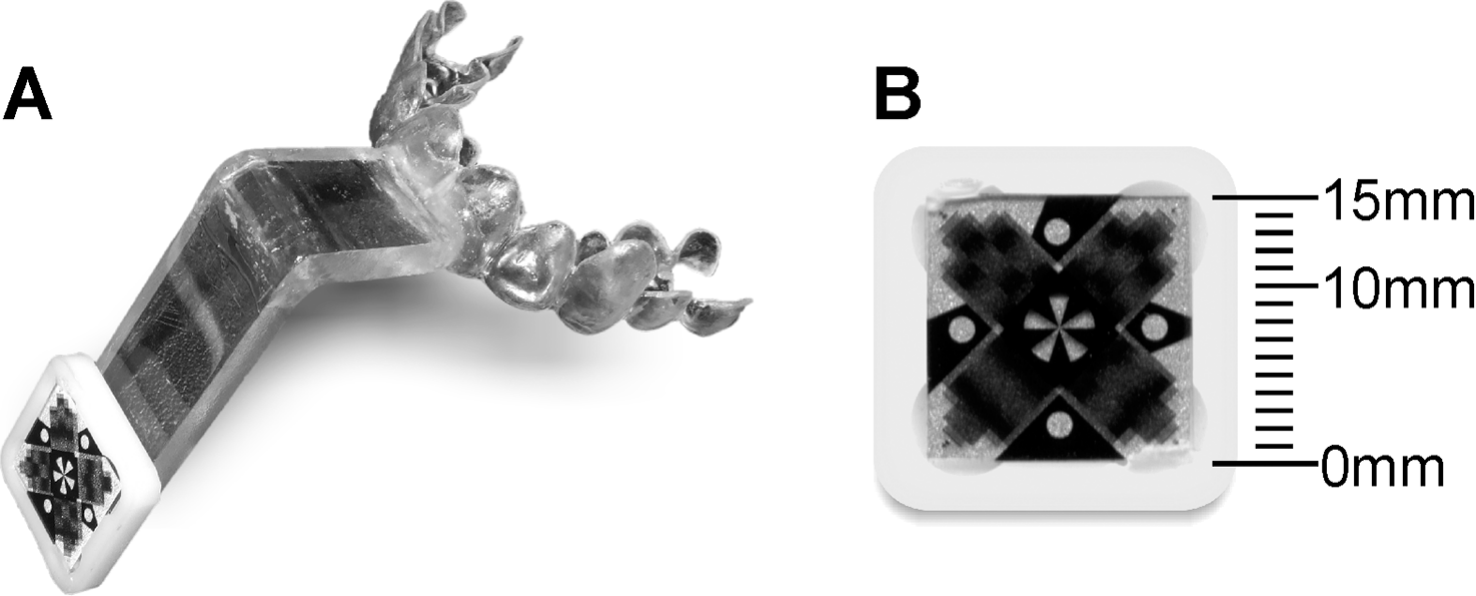
Dental brace and MPT marker. An individually manufactured dental brace (A) was worn by the subject during the measurement. A passive marker similar to the one shown in (B) was mounted on the brace’s extension reaching out of the helmet-design head coil. Planar grating patterns were printed on both sites of a transparent substrate forming moiré patterns. The retro reflective background of the marker ensured visibility to the camera and lighting unit (CLU) at low light exposure levels.

**Fig 2:**
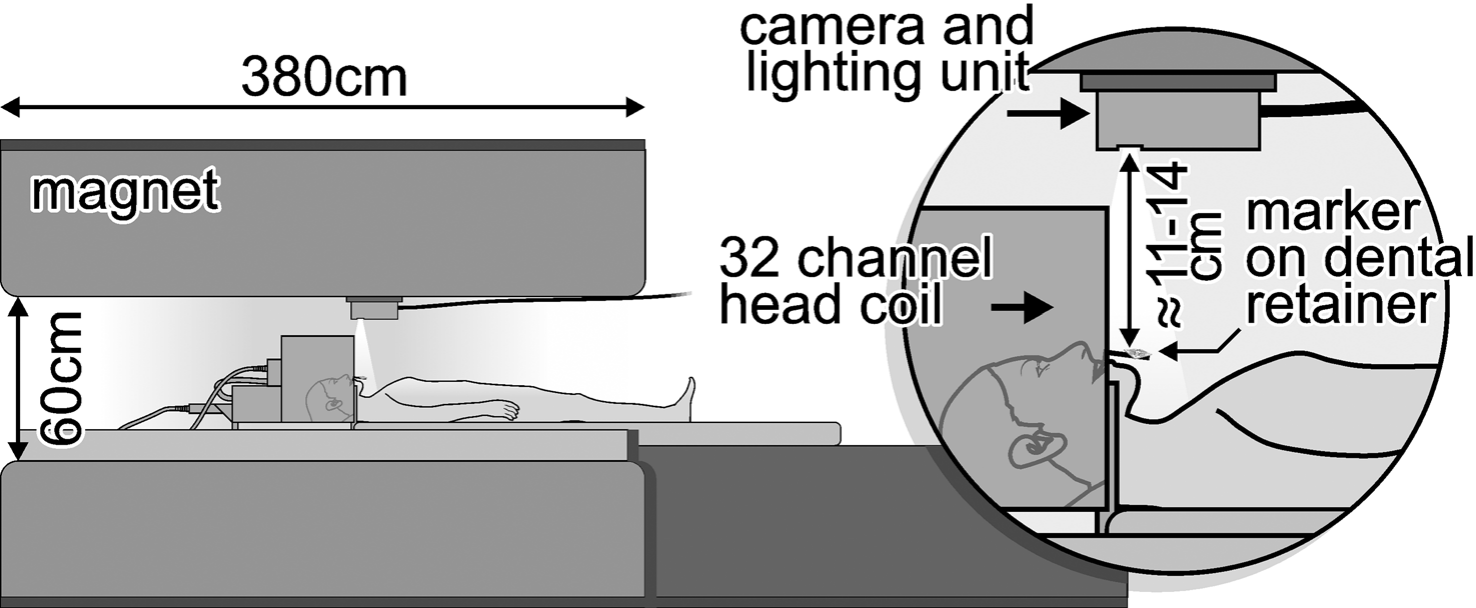
Experimental setup for motion tracking during the MRI measurements. The tracking systems CLU was mounted on the inner cover inside the magnet bore. The MPT marker was presented at a distance of ≈ 11–14 cm from the camera on the extension of the dental brace reaching out of the 32-channel helmet-design head coil.

PMC was used in the acquisition of the images presented in this work. The subjects’ motion was optically tracked using a moiré phase tracking (MPT) system [37, 39]. This technology allows for high precision tracking of out-of-plane rotations, by deriving pose information from changes in a moiré pattern visible on a 15mm marker (Fig 1). Moreover, through photogrammetric techniques and image processing, the marker position could be tracked along six degrees of freedom. In previous studies [39] this system was described in detail and was used successfully to acquire motion corrected MRI images, particularly of intentionally moving subjects, at different field strengths with resolutions of up to 0.3 × 0.3 × 3 mm. In brief, the camera and lighting unit (CLU) of the MPT system consisted of a customized fixed focus, fixed aperture VGA camera (656 × 492 pixels) and LED flash illumination compatible with the high-field environment. The camera is mounted over the subject’s head inside the magnet bore using adhesive tape (Fig 2). Proper care was taken to make this mounting stable and vibration free. The photogrammetrically pre-calibrated camera [40] tracked the subject with 60 frames per second (fps) (maximum sampling rate is 85Hz).

### Cross calibration

Cross-calibration was performed between the tracking system and the scanner coordinates as described in [41]. In this method, a rigid body motion model is assumed for the cross-calibration. Test object motion detected with the MPT system and with MRI allows for the determination of a coordinate transformation between the camera and scanner coordinates [33].

### Marker fixation

The marker must be attached rigidly to the subject’s head to accurately represent the motion of the human brain. Any deviation of the marker motion from the head motion will reduce the accuracy of the applied correction. Fixing the marker onto the forehead or between the eyes of the subject is very convenient but the detected motion may differ from the true motion of the head due to the flexibility of the skin. Herbst et al. showed that marker fixation on the skin using tape or modeling clay is not suitable for high resolution scanning and motion artifacts can be seen [42]. They demonstrated the superiority of a mouthpiece for best stability. Therefore, a removable dental brace was custom-made for each subject to fix the marker (Fig 1). An extension of the brace reaches out of the helmet-design head coil and allows a line of sight from the camera to the marker. The distance between the camera and the marker attached to the extension was approximately 11–14 cm (Fig 2). The brace was attached to the teeth of the upper jaw without contact to the gum, to avoid motion caused by soft tissue. The MPT marker attached to this dental brace was tracked by the camera at 60 frames per second. No filtering/smoothing or other post processing was applied to the tracking data.

### In vivo scans

All experiments on human volunteers were performed with the approval of the ethics committee of the Otto-von-Guericke University Magdeburg, Germany. Written informed consent was obtained from all subjects prior to the scans. Four healthy and co-operative volunteers (including two from the list of authors) with prior experience in motion correction experiments and in possession of individually created dental braces were recruited.

All scans were carried out on a 7 T whole body MRI (Siemens Medical Solutions, Erlangen, Germany) equipped with a 32-channel head coil (Nova Medical, Wilmington, MA, USA). The volunteers were instructed to remain as stationary as possible during the measurement. The marker was attached to the mouth piece and the 6-degrees-of-freedom motion data was logged for analysis and comparison.

Sequences were modified to include the functionality of online gradient and frequency changes to update the imaging volume during scan execution using the real time tracking data from the MPT system [33]. The FoV was adjusted for every k-space line (once per TR) using the most current motion information. Except for the integration of PMC, the vendor’s original sequences were used. If necessary the limit for the matrix size was expanded.

We acquired high resolution data with PMC using a T2*-weighted 2D gradient echo sequence, a T1-weighted 3D MPRAGE and a 3D ToF sequence (throughout this paper, these scans are referred to as “highest resolution scans”). Because of the very long acquisition times, it was not possible to repeat all scans under different conditions, i.e. without motion correction. Thus no comparison images were acquired for the highest resolution images. Scans with lower resolutions, and / or less averages and thus shorter acquisition times were acquired with PMC enabled and disabled (referred to as “comparison scans”). For 3D-ToF GRAPPA acceleration factor 2 was used to fit a single average into the reconstruction memory. We chose the bandwidth to balance between SNR and ADC duration-related PSF broadening. ADC duration was kept smaller than the T2* relaxation times of brain tissues leading to minimal additional blurring in the readout direction. No resulting artifacts were visible. The scan parameters for the comparison scans and for the highest resolution scans, as well as all scan times of the individual scans are depicted in Table 1. All the images were processed with online gradient distortion correction but no further post processing steps were applied to these images.

**Table 1:** In vivo MRI scans: scan parameters.

	comparison scans		highest resolution scans		
Sequence	3D MPRAGE	T2* 2D GRE	T2* 2D GRE	3D MPRAGE	3D ToF
motion correction	on / off	on / off	on	on	on
resolution (mm)	0.44 iso	0.25 × 0.25 × 2.0	0.12 × 0.12 × 0.6	0.44 iso	0.2 iso
Matrix size (voxel)	464 × 464	756 × 896	1690 × 1744	464 × 464	744 × 992
voxel volume (mm^3^)	0.0852	0.125	0.0087	0.0852	0.008
slices	384	28	21	384	140
averages	1	2	2	2	2
TR (ms)	2600	900	900	2600	24
TE (ms)	2.3	18	17.7	2.3	6.52
TI (ms)	1250			1250	
flip angle (deg)	5	45	35	5	19
bandwidth (Hz/px)	251	60	49	251	120
total ADC duration (ms)	3.98	16.7	20.4	3.98	8.3
TA (min:sec, per Avg.)	20:12	11:22	25:26	20:12	28:15
TA (min:sec, all Avg.)	20:12	22:45	50:52	40:24	56:30
parallel imaging	-	-	-	-	GRAPPA 2

Scan parameters for the GRE and MPRAGE comparison scans with and without motion correction and for the highest resolution GRE, MPRAGE and ToF scans with motion correction.

## Results

### Comparison study

**Fig 3:**
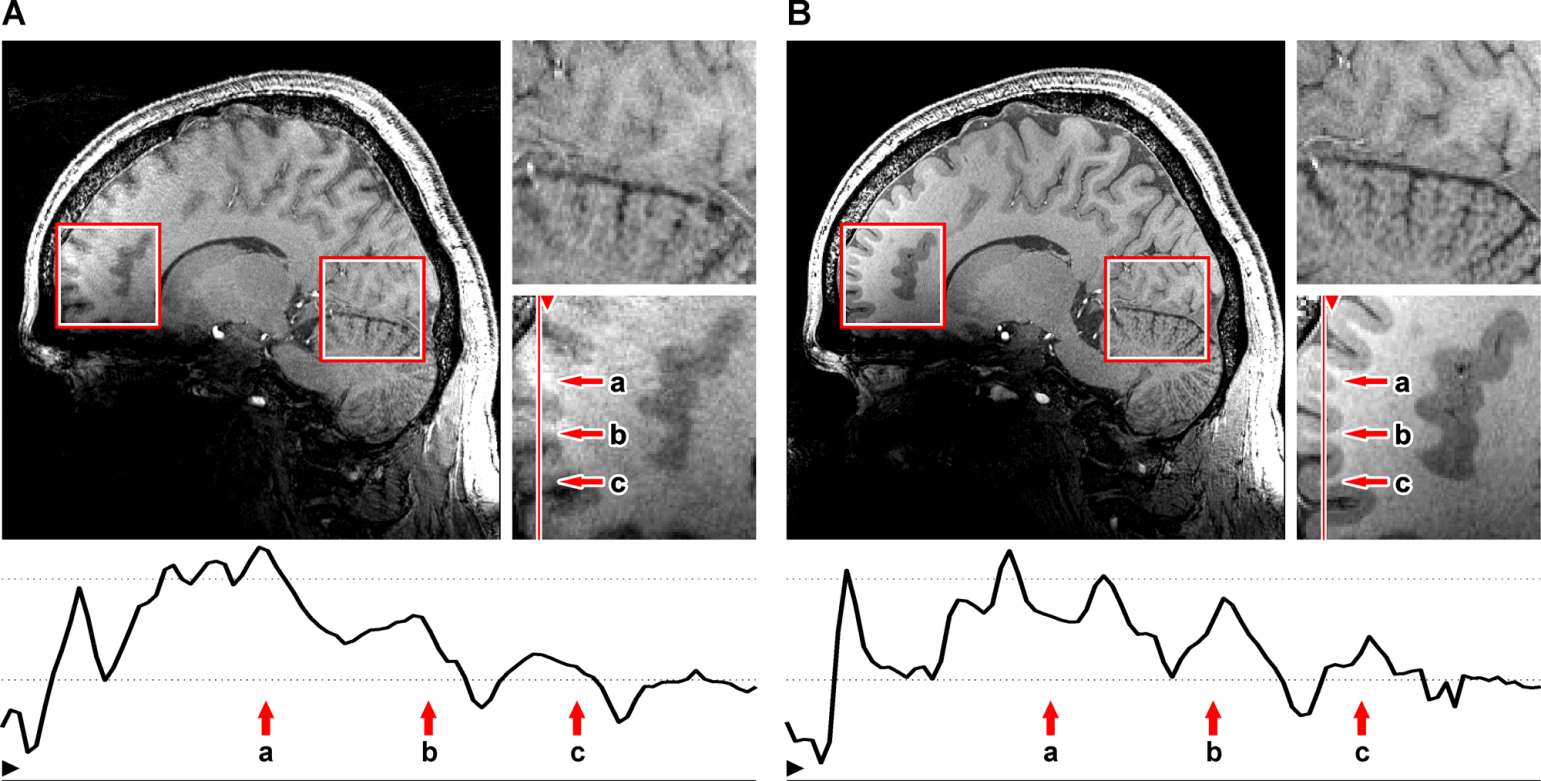
MPRAGE comparison scans. 0.44 mm isotropic 3D MPRAGE with motion correction off (A) and on (B) and magnifications of the marked regions. The plot shows the signal intensity profile of the line marked in the magnifications. Image-degrading effects caused by motion artifacts include noise amplification and creation of bogus structures e.g. doubled borders (a), blurring of structures such as flattening of signal intensity gradients (b) and plateaus (c).

**Fig 4:**
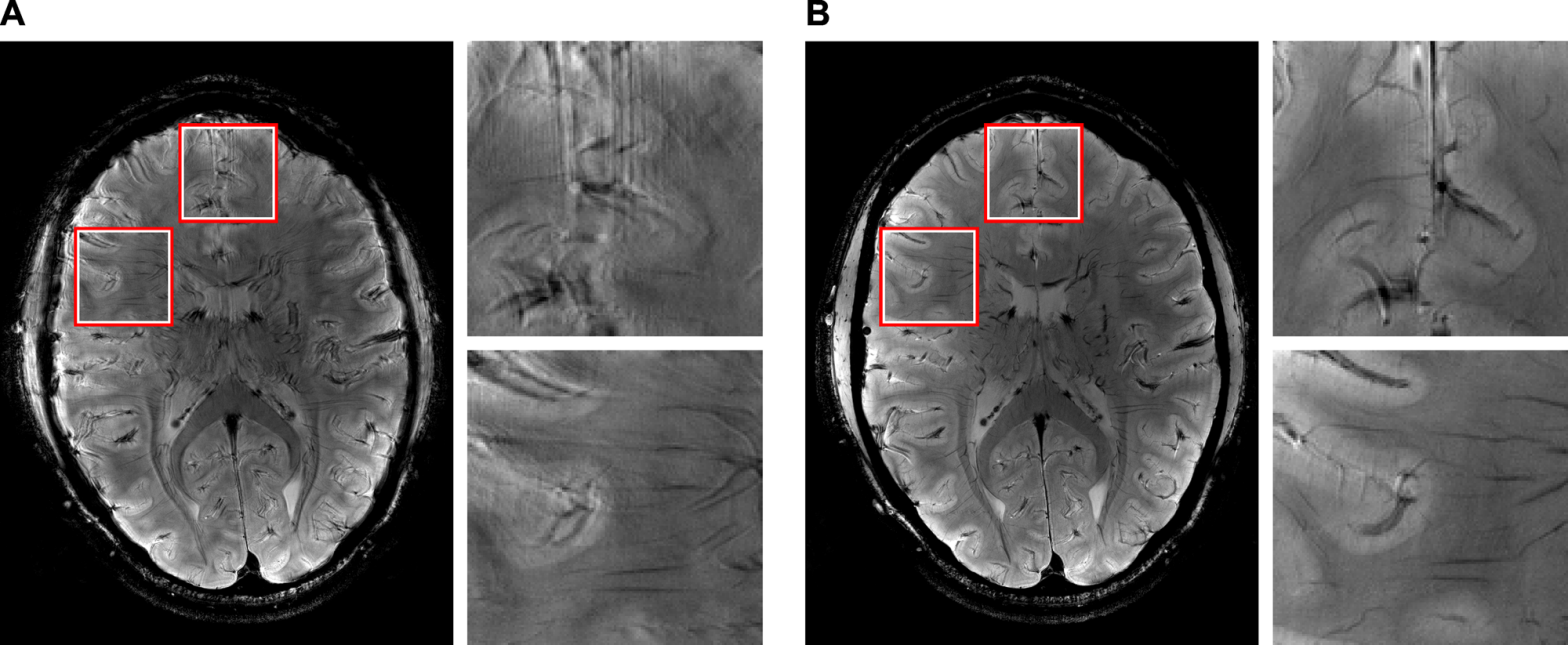
GRE comparison scans. 0.25 × 0.25 × 2 mm gradient echo with motion correction off (A) and on (B) and magnifications of the marked regions.

**Fig 5:**
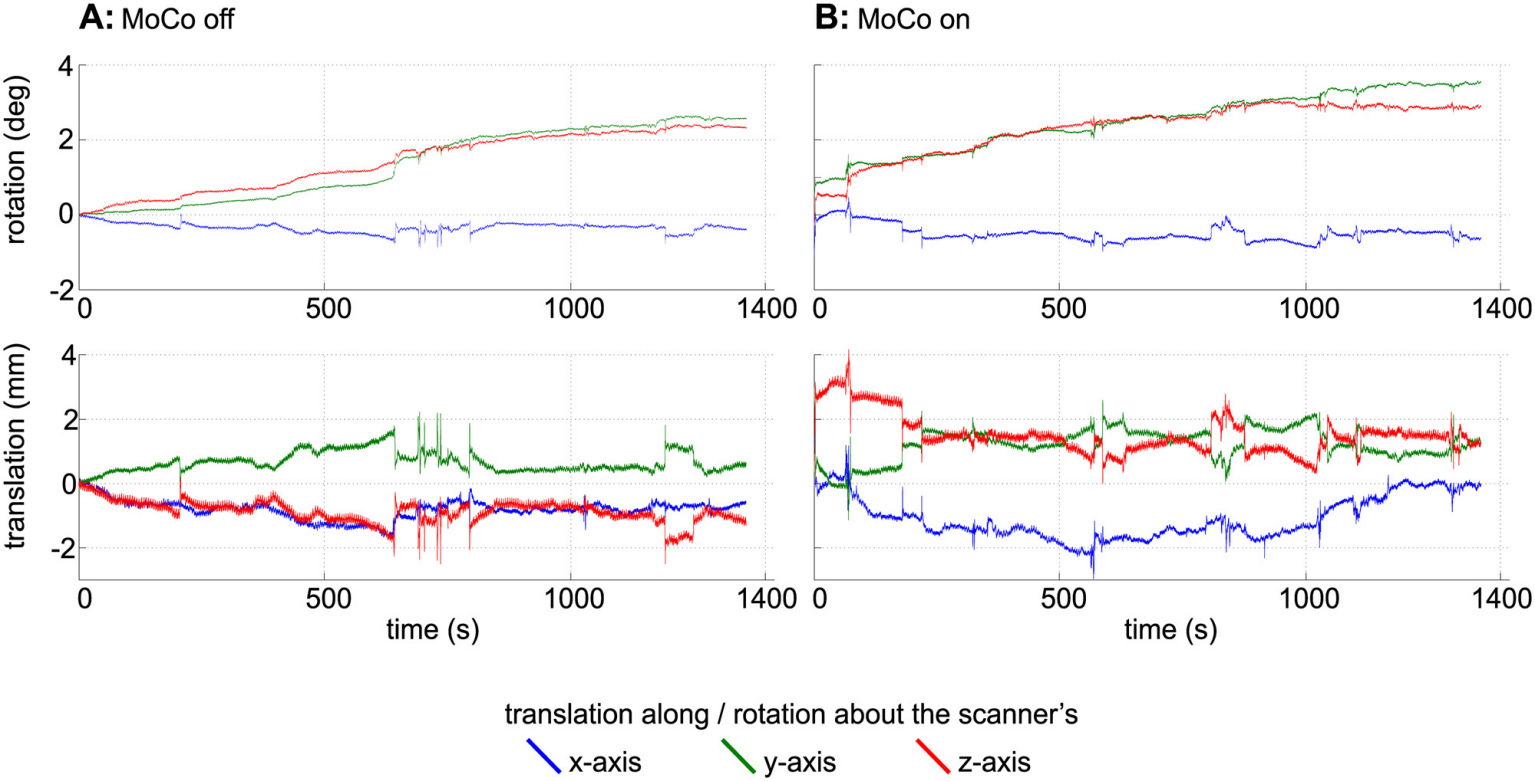
Motion plots for the GRE comparison scans. The plots show the translations and rotations recorded during the GRE scans (Fig 4) without (A) and with (B) motion correction. For this plot, the data delivered by the MPT tracking system were converted to scanner coordinates.

Slice by slice comparison of the corrected and uncorrected MPRAGE images of 0.44mm isotropic resolution (Fig 3) and the corrected and uncorrected 0.25 × 0.25 × 2 mm gradient echo images (Fig 4) reveals the superior quality of the data acquired with motion correction. The images acquired without motion correction show significant motion artifacts and blurring. An intensity plot on the frontal area of the MPRAGE data reveals motion artifacts in the image acquired without PMC (red lines and plots in Fig 3). These artifacts include doubled or blurred edges creating bogus structures, flattened signal intensity gradients and plateaus, reduced intensity peaks and increased noise. The motion plots for the GRE scans (Fig 5) show a similar amount of motion for both scans with slightly more motion in the corrected scan as depicted in Table 2. The motion corrected GRE images show small details such as very small vessels and cortical layers; the motion corrected MPRAGE images show well defined borders between grey and white matter or single folia in the cerebellum, only hardly visible in the uncorrected images (see magnification in Figs 3 and 4).

**Table 2:** In vivo MRI scans: amount of motion during the scans.

	comparison scans				highest resolution scans		
	3D MPRAGE		T2* 2D GRE		T2* 2D GRE	3D MPRAGE	3D ToF
	MoCo off	MoCo on	MoCo off	MoCo on			
std.dev.							
X-Trans.	0.94	1.5	0.30	0.67	0.94	0.61	0.19
Y-Trans.	1.4	1.1	0.32	0.50	0.40	0.90	0.71
Z-Trans.	0.93	1.7	0.35	0.60	0.63	0.52	1.3
X-Rot.	0.56	0.53	0.13	0.22	0.21	0.36	0.37
Y-Rot.	0.28	0.24	0.96	0.78	0.43	0.18	0.10
Z-Rot.	0.07	0.31	0.76	0.70	0.12	0.49	0.09
abs.range							
X-Trans.	5.6	11.9	1.9	4.5	4.1	2.5	3.8
Y-Trans.	18.1	7.9	2.4	4.1	1.9	4.8	5.6
Z-Trans.	17.6	9.6	2.8	4.2	2.3	4.2	8.1
X-Rot.	7.9	3.6	0.94	1.6	0.82	1.8	2.9
Y-Rot.	1.2	3.7	2.7	3.1	1.9	0.99	1.1
Z-Rot.	1.5	2.5	2.5	3.4	0.51	2.1	1.2

Standard deviation and absolute range of the X-, Y- and Z- translations and rotations measured by the tracking system for all in vivo scans.

### Highest resolution images

**Fig 6:**
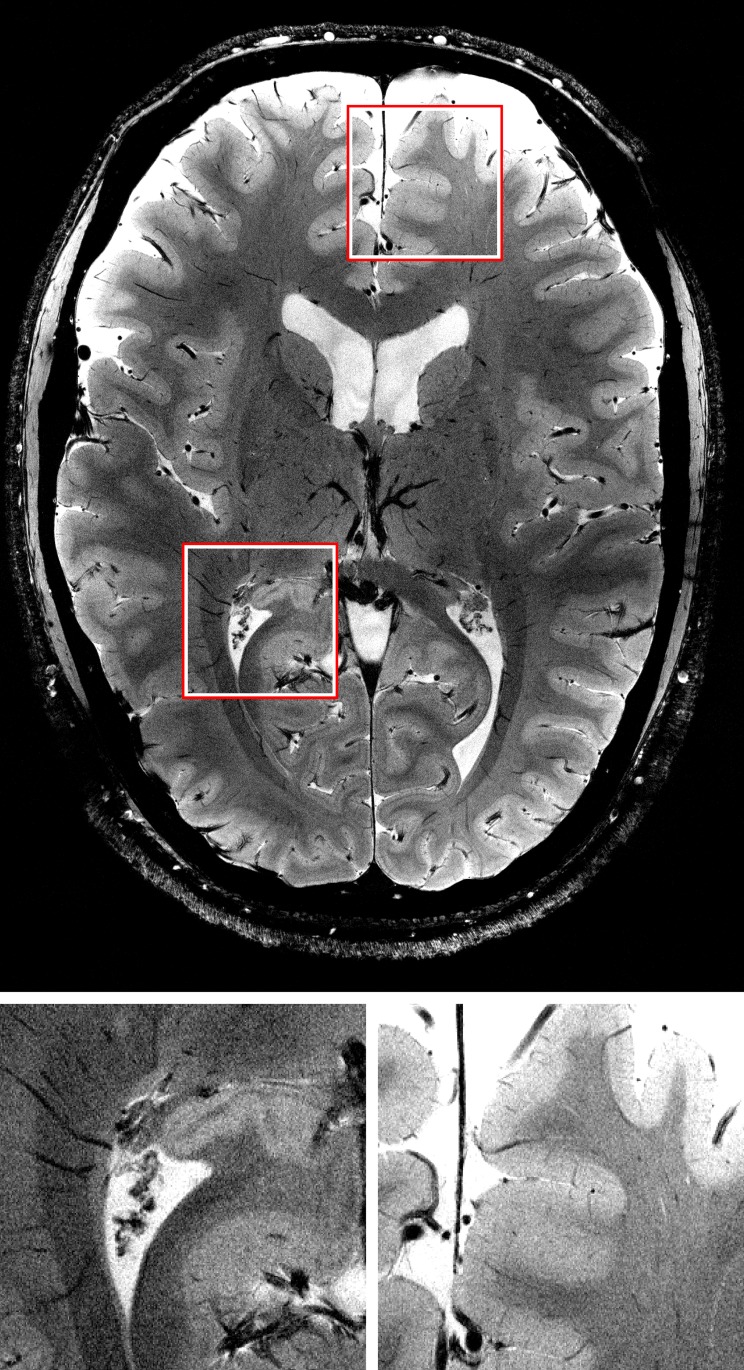
One slice of the highest resolution GRE scan. At a resolution of 0.12 × 0.12 × 0.6 mm structures of one to two pixel in width are identifiable and clearly defined. Magnifications of the marked regions are shown below. See S1 Video for full data set.

**Fig 7:**
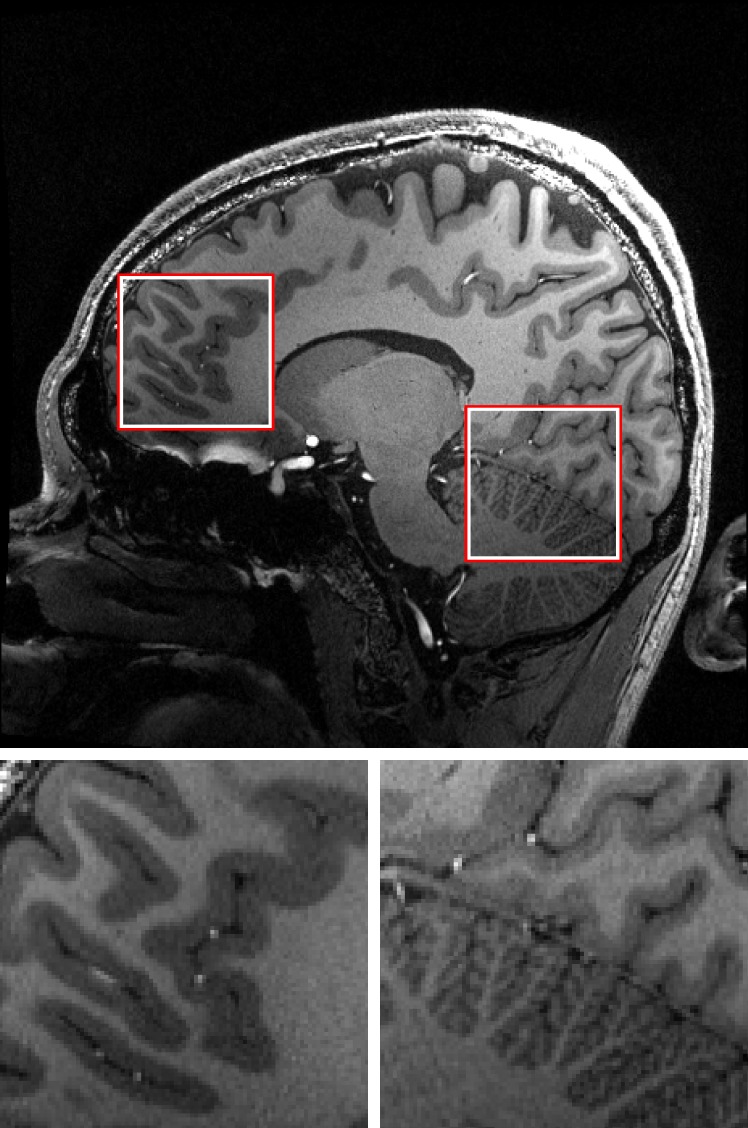
One slice of the highest resolution MPRAGE scan. Note the sharpness in small structures such as vessels and the folia in the cerebellum in this 0.44 mm isotropic 3D MPRAGE data. Magnifications of the marked regions are shown below. See S2 Video for full data set.

**Fig 8:**
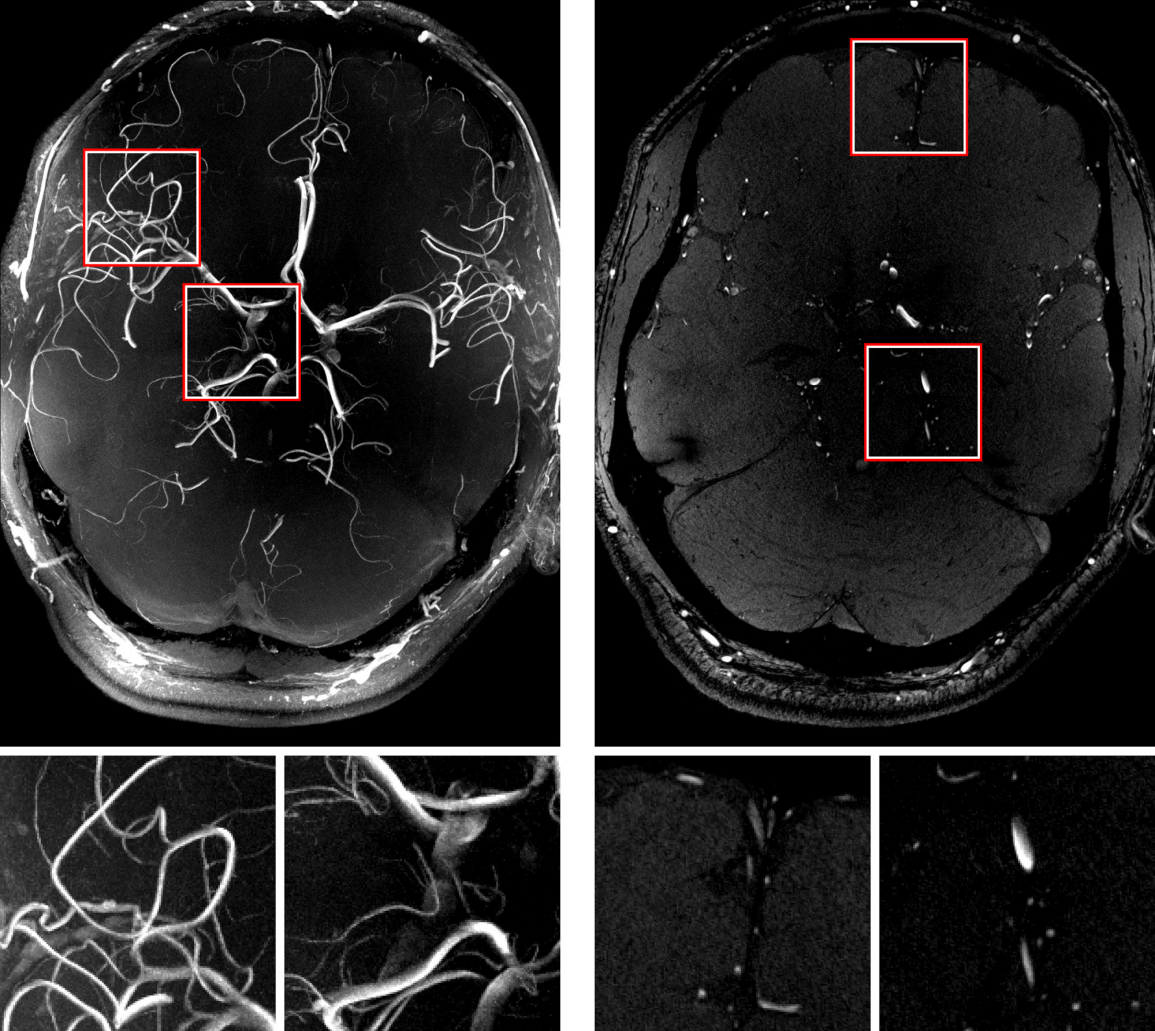
A maximum intensity projection (MIP) of the highest resolution time-of-flight scan (left) and one slice of the dataset (right). This 0.2 mm isotropic dataset shows five to seven bifurcations of the middle cerebral artery for the vessels in the FoV which covers only 2.8cm through-plane and does not include the full brain’s vessel geometry. Magnifications of the marked regions are shown below. See S3 Video for full data set.

**Fig 9:**
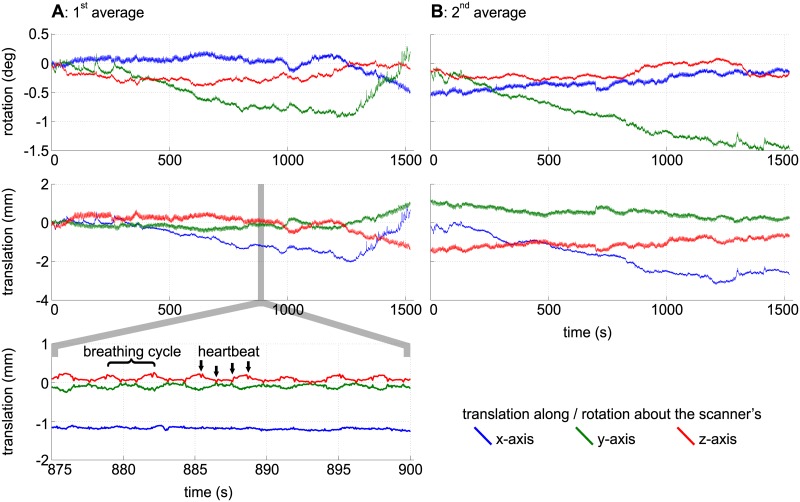
Motion plots for the highest resolution GRE scans. The plots show translations and rotations recorded during the first average (A) and the second average (B) of the highest resolution GRE scan (Fig 6). Motion caused by breathing and heartbeat is clearly visible in the closeup.

The highest resolution GRE (Fig 6 and S1 Video), 3D MPRAGE (Fig 7 and S2 Video) and TOF (Fig 8 and S3 Video) images also show small structures like tissue borders or vessels with high degree of detail. In the GRE image, structures of 1–2 pixels (0.12–0.24 mm) in width are clearly visible. The amount (absolute range and standard deviation) of translational and rotational motion during the scans is given in Table 2. The complete motion data delivered by the tracking system during the GRE measurements and converted to scanner co-ordinates are shown in Fig 9. The data show that the MPT tracking system is sensitive and accurate enough to detect microscopic physiological motion from breathing and heartbeat.

Multiple averages ensured higher SNR in the images but resulted in very long scan times. The SNR was calculated by dividing the mean of a relatively small region (24–3222 pixels) in the specific tissue with visually homogeneous signal intensities by the standard deviation of a relatively large (8206–357654 pixels) region in the background outside the object without visible artifacts. For each tissue, this calculation was performed for three different locations in three slices. The mean values of the three calculations for each tissue are given in Table 3.

**Table 3:** In vivo MRI scans: SNR analysis.

T2* 2D GRE		3D MPRAGE		3D ToF	
white matter	≈ 11	white matter	≈ 58	arteries	≈ 47
grey matter	≈ 22	grey matter	≈ 46		
CSF	≈ 32	CSF	≈ 18		

Approximate SNR values calculated by dividing the mean of a relatively small region in a specific tissue. By the standard deviation of a relatively large region in the background without visible artifacts.

## Discussion

In this study we showed that the increased SNR at 7 T can be used to increase image-resolution and visualize very small and fine structures such as cortical layers and very small vessels of the brain. Despite the higher field strength, multiple averages are necessary to obtain images of sufficiently high quality. Especially the GRE images shown may benefit from even more averages to further reduce image noise. We present in vivo data with effective resolutions which are very difficult if not impossible to acquire without motion correction during very long scans. PMC was used with an external optical tracking system to compensate for involuntary and inevitable motion of the head caused by breathing, heartbeat and muscle relaxation. Thereby ultra high-resolution brain images on well trained volunteers were acquired. The actual resolution of the images shown in this study depends on the sequence (see Table 1) and is mainly limited by subject discomfort caused by the long scan times. If larger image matrices are acquired, off-line reconstruction is required due to memory limitations of the scanner’s image reconstruction hardware. The scan times including positioning and pre-scans were about 90 min for each very high resolution dataset. Even though these studies were performed on well-trained volunteers who can hold their head still for a long time, physiological motion was unavoidable. Maclaren et al. showed that the correction of this motion leads to improved image quality for sub-millimeter resolutions [39]. They conclude that the benefit for the effective resolution from motion correction, i.e. reduction of motion-induced blurring, is higher for very high resolution images. These findings correspond well to our observations in this comparison study. The image quality of the corrected scans is superior to the uncorrected data and visually free of motion artifacts, even though the motion was slightly stronger than in the uncorrected data. Due to the very long scan times, no uncorrected comparison images were acquired for the highest resolution images. However, the benefit of motion correction visible in the 0.25 × 0.25 × 2 mm gradient echo images and the 0.44 iso MPRAGE images of the comparison study suggests, that the improvement achieved by motion correction is even more dramatic for higher resolutions, as smaller voxel sizes are susceptible to even smaller movements. Thus we expect the motion artifacts to be stronger for uncorrected versions of the highest resolution data, as the amount of motion was similar to the comparison scans.

Several other groups have successfully applied PMC using external tracking on healthy volunteers. The goal of most of these studies was to show the potential benefit of PMC for scans with patients prone to cause motion artifacts such as children or patients suffering from Parkinson’s disease. Accordingly, the volunteers were instructed to move their head during image acquisition and the resolution of the images acquired were not as high as we have reported in this study. Herbst et al. also investigated the impact of physiological motion (cardiac, breathing or involuntary muscle relaxation) on artifact strength [20]. Their data suggest, that long term drift caused by muscle relaxation has the strongest impact. In addition, they comment that the ability of a motion correction system to correct for short term (fast) motion depends on the time needed for processing the position data in the correction pipeline. The latency of the prospective corrections is 20–30 ms, depending on the selected framerate and marker visibility. According to Maclaren et al., the accuracy of the tracking system should be five to ten-fold higher than the nominal voxel edge length [17]. Considering this, the applied tracking system is sufficiently fast to correct for motion in the velocity range of 0.4 to 2.2 mm/s for the resolutions used in this study. For that reason, experienced subjects were selected for the experiments. The motion data shows the largest motion amplitude in long term drift effects, i.e. in slow motion. Due to the latency, very fast motion such as coughing would not be fully corrected and may thus create residual motion artifacts. Besides latency, spatial accuracy of the tracking system is a further challenge in PMC. The accuracy depends heavily on the marker attachment to the head of the subject. Herbst et al. suggested dental braces for very high resolution imaging [42]. In this study we used custom-made dental braces which can be fixed tightly to the upper front teeth of the subject. The braces were designed so that there was no contact with the lower jaw and soft tissues even when the mouth is closed. This ensured a rigid attachment to the head and thus it delivered the most accurate motion data representative of the brain’s motion, where periodic motion caused by breathing and heartbeat in the range of below 0.07mm is clearly visible. Even though fixing the marker on the dental brace delivers accurate tracking data, it may cause slight discomfort in some subjects. Moreover the subject is required to visit a dentist prior to the measurement to create the customized braces. A more comfortable, less time consuming marker fixation method will be required if PMC is applied in a clinical setting. As very high resolutions are not required in clinical routine, a more comfortable marker fixation using tape or clay might offer a sufficiently good compromise between accuracy of the tracking data and patient comfort [42].

The external tracking system only records the head’s motion. Thus, residual non-rigid motion within the skull such as brain pulsation or eye-movement, cannot be corrected. Eye movement can cause visible and disturbing artifacts in otherwise unaffected areas of the brain, this effect can be avoided by choosing an appropriate direction for phase encoding. Artifacts caused by brain pulsation have not been observed in our study. Thus, for the resolution used in our study, (involuntary) subject motion is the main limiting factor. Whether higher resolution will be limited by system precision, non-rigidity of the brain/head or by other factors (e.g. the mounting of the MPT marker or the rigidity of the dental brace) remains to be studied.

To further increase the spatial image resolution and SNR, more averages will be necessary. As subject discomfort does not allow for measurements longer than 90–120 min, splitting the scan to several sessions could offer an option to acquire the data for total scan times in the range of several hours. We showed that repositioning of the dental brace is possible with an accuracy of better than 1mm [43]. For very high resolution imaging a better repositionable fixation would be needed or further registration of the datasets would be necessary before combination.

## Conclusion

The effective resolution of in vivo MRI can be increased substantially using PMC, but long scan times and high fields are necessary to gain enough signal. Thus, in addition to the capabilities of state of the art hardware, it is the subjects’ patience and their ability to maintain the same pose for prolonged periods, which defines the achievable resolution. This is especially true for neuroscience and biology applications, where a great interest in very high resolution images exists. To the best of our knowledge, the very high resolution images shown in this study are amongst the highest, if not the highest resolutions of in vivo human brain MRI ever acquired.

## Supporting Information

S1 Video Video of highest resolution GRE data. Full dataset (21 slices) of the highest resolution T2* 2D GRE scan (shown in Fig 6).
(ZIP)

S2 Video Video of highest resolution MPRAGE data. Full dataset (384 slices) of the highest resolution 3D MPRAGE scan (shown in Fig 7).
(ZIP)

S3 Video Video of highest resolution ToF data. Full dataset (140 slices) of the highest resolution 3D ToF scan (shown in Fig 8).
(ZIP)

## References

[1] Pohmann R, Speck O, Scheffler K. Signal-to-noise ratio and MR tissue parameters in human brain imaging at 3, 7, and 9.4 Tesla using current receive coil arrays. Magn Reson Med. 2015; 10.1002/mrm.25677 25820458

[2] Lüsebrink F, Wollrab A, Speck O. Cortical thickness determination of the human brain using high resolution 3 T and 7 T MRI data. NeuroImage. 2013;70:122–131. 10.1016/j.neuroimage.2012.12.016 23261638

[3] Yang S, Kanowski M, Fischer K, Godenschweger F, Zhong K, Walter M, et al ULtra-High Resolution Post-Mortem 3D Imaging of the Entire Human Brain at 7T. MAGMA. 2011;24(Supplement 1):490–491.

[4] Yang S, Yang Z, Fischer K, Zhong K, Stadler J, Godenschweger F, et al Integration of Ultra-High Field MRI and Histology for Connectome Based Research of Brain Disorders. Front Neuroanat. 2013;7(31). 10.3389/fnana.2013.00031 PMC3784919 24098272

[5] Ge Y, Zohrabian VM, Grossman RI. Seven-Tesla magnetic resonance imaging: new vision of microvascular abnormalities in multiple sclerosis. Archives of neurology. 2008;65(6):812–816. 10.1001/archneur.65.6.812 18541803 PMC2579786

[6] Christoforidis GA, Yang M, Abduljalil A, Chaudhury AR, Newton HB, McGregor JM, et al “Tumoral pseudoblush” identified within gliomas at high-spatial-resolution ultrahigh-field-strength gradient-echo MR imaging corresponds to microvascularity at stereotactic biopsy. Radiology. 2012;264(1):210–217. 10.1148/radiol.12110799 22627600 PMC3380412

[7] Kirov II, Hardy CJ, Matsuda K, Messinger J, Cankurtaran CZ, Warren M, et al In vivo 7 Tesla imaging of the dentate granule cell layer in schizophrenia. Schizophr Res. 2013;147(2–3):362–367. 10.1016/j.schres.2013.04.020 23664589 PMC3709603

[8] Stamm AC, Wright CL, Knopp MV, Schmalbrock P, Heverhagen JT. Phase contrast and time-of-flight magnetic resonance angiography of the intracerebral arteries at 1.5, 3 and 7 T. Magn Reson Imaging. 2013;31(4):545–549. 10.1016/j.mri.2012.10.023 23219250

[9] Hanke M, Baumgartner FJ, Ibe P, Kaule FR, Pollmann S, Speck O, et al A high-resolution 7-Tesla fMRI dataset from complex natural stimulation with an audio movie. Scientific data. 2014;1:140003 10.1038/sdata.2014.3 25977761 PMC4322572

[10] Wrede KH, Johst S, Dammann P, Özkan N, Mönninghoff C, Kraemer M, et al Improved cerebral time-of-flight magnetic resonance angiography at 7 Tesla–feasibility study and preliminary results using optimized venous saturation pulses. PloS one. 2014;9(9):e106697 10.1371/journal.pone.0106697 25232868 PMC4169393

[11] Forstmann BU, Keuken MC, Schafer A, Bazin PL, Alkemade A, Turner R. Multi-modal ultra-high resolution structural 7-Tesla MRI data repository. Sci Data. 2014;1:140050 10.1038/sdata.2014.50 25977801 PMC4421933

[12] Keuken MC, Bazin PL, Schäfer A, Neumann J, Turner R, Forstmann BU. Ultra-high 7T MRI of structural age-related changes of the subthalamic nucleus. J Neurosci. 2013;33(11):4896–4900. 10.1523/JNEUROSCI.3241-12.2013 23486960 PMC6619019

[13] Budde J, Shajan G, Scheffler K, Pohmann R. Ultra-high resolution imaging of the human brain using acquisition-weighted imaging at 9.4T. NeuroImage. 2014;86:592–598. 10.1016/j.neuroimage.2013.08.013 23954486

[14] Zaitsev M, Maclaren J, Herbst M. Motion artifacts in MRI: A complex problem with many partial solutions. J Magn Reson Imaging. 2015;p. n/a–n/a. Available from: 10.1002/jmri.24850. 10.1002/jmri.24850 PMC4517972 25630632

[15] Stucht D, Yang S, Schulze P, Danishad A, Kadashevich I, Bernarding J, et al Improved Image Segmentation with Prospective Motion Correction in MRI In: Tolxdorff T, Deserno TM, Handels H, Meinzer HP, editors. Bildverarbeitung für die Medizin 2012. Informatik aktuell. Springer Berlin Heidelberg; 2012 p. 27–32.

[16] Reuter M, Tisdall MD, Qureshi A, Buckner RL, van der Kouwe, André J W, Fischl B. Head Motion During MRI Acquisition Reduces Gray Matter Volume and Thickness Estimates. NeuroImage. 2015;107:107–115. 10.1016/j.neuroimage.2014.12.006 25498430 PMC4300248

[17] Maclaren J, Speck O, Stucht D, Schulze P, Hennig J, Zaitsev M. Navigator Accuracy Requirements for Prospective Motion Correction. Magn Reson Med. 2010;63(1):162–170. 10.1002/mrm.22191 19918892 PMC2933924

[18] Schultz CL, Alfidi RJ, Nelson AD, Kopiwoda SY, Clampitt ME. The Effect of Motion on Two-Dimensional Fourier Transformation Magnetic Resonance Images. Radiology. 1984;152(1):117–121. 10.1148/radiology.152.1.6729101 6729101

[19] Wood ML, Henkelman RM. MR Image Artifacts from Periodic Motion. Med Phys. 1985;12(2):143–151. 10.1118/1.595782 4000069

[20] Herbst M, Maclaren J, Lovell-Smith C, Sostheim R, Egger K, Harloff A, et al Reproduction of Motion Artifacts for Performance Analysis of Prospective Motion Correction in MRI. Magn Reson Med. 2014;71(1):182–190. 10.1002/mrm.24645 23440737 PMC3674114

[21] Fletcher BD, Jacobstein MD, Nelson AD, Riemenschneider TA, Alfidi RJ. Gated Magnetic Resonance Imaging of Congenital Cardiac Malformations. Radiology. 1984;150(1):137–140. 10.1148/radiology.150.1.6689753 6689753

[22] Lanzer P, Botvinick EH, Schiller NB, Crooks LE, Arakawa M, Kaufman L, et al Cardiac Imaging Using Gated Magnetic Resonance. Radiology. 1984;150(1):121–127. 10.1148/radiology.150.1.6227934 6227934

[23] Runge VM, Clanton JA, Partain CL, James AE. Respiratory Gating in Magnetic Resonance Imaging at 0.5 Tesla. Radiology. 1984;151(2):521–523. 10.1148/radiology.151.2.6709928 6709928

[24] Axel L, Summers RM, Kressel HY, Charles C. Respiratory effects in two-dimensional Fourier transform MR imaging. Radiology. 1986;160(3):795–801. 10.1148/radiology.160.3.3737920 3737920

[25] Versluis MJ, Peeters JM, van Rooden S, van der Grond J, van Buchem M A, Webb AG, et al Origin and reduction of motion and f0 artifacts in high resolution T2*-weighted magnetic resonance imaging: application in Alzheimer’s disease patients. NeuroImage. 2010;51(3):1082–1088. 10.1016/j.neuroimage.2010.03.048 20338252

[26] Versluis MJ, Sutton BP, de Bruin P W, Börnert P, Webb AG, van Osch M J. Retrospective image correction in the presence of nonlinear temporal magnetic field changes using multichannel navigator echoes. Magn Reson Med. 2012;68(6):1836–1845. 10.1002/mrm.24202 22362637

[27] Ehman RL, McNamara MT, Pallack M, Hricak H, Higgins CB. Magnetic Resonance Imaging with Respiratory Gating: Techniques and Advantages. AJR Am J Roentgenol. 1984;143(6):1175–1182. 10.2214/ajr.143.6.1175 6333787

[28] Pipe JG. Motion correction with PROPELLER MRI: Application to Head Motion and Free-Breathing Cardiac Imaging. Magn Reson Med. 1999;42(5):963–969. 10.1002/(SICI)1522-2594(199911)42:5<963::AID-MRM17>3.0.CO;2-L 10542356

[29] Ward HA, Riederer SJ, Grimm RC, Ehman RL, Felmlee JP, Jack CR. Prospective Multiaxial Motion Correction for fMRI. Magn Reson Med. 2000;43(3):459–469. 10.1002/(SICI)1522-2594(200003)43:3<459::AID-MRM19>3.0.CO;2-1 10725890

[30] Lee CC, Grimm RC, Manduca A, Felmlee JP, Ehman RL, Riederer SJ, et al A Prospective Approach to Correct for Inter-Image Head Rotation in fMRI. Magn Reson Med. 1998;39(2):234–243. 10.1002/mrm.1910390210 9469706

[31] Ooi MB, Krueger S, Muraskin J, Thomas WJ, Brown TR. Echo-Planar Imaging with Prospective Slice-by-Slice Motion Correction Using Active Markers. Magn Reson Med. 2011;66(1):73–81. 10.1002/mrm.22780 21695720 PMC3122130

[32] Ooi MB, Krueger S, Thomas WJ, Swaminathan SV, Brown TR. Prospective Real-Time Correction for Arbitrary Head Motion Using Active Markers. Magn Reson Med. 2009;62(4):943–954. 10.1002/mrm.22082 19488989 PMC3033410

[33] Zaitsev M, Dold C, Sakas G, Hennig J, Speck O. Magnetic Resonance Imaging of Freely Moving Objects: Prospective Real-Time Motion Correction Using an External Optical Motion Tracking System. NeuroImage. 2006;31(3):1038–1050. 10.1016/j.neuroimage.2006.01.039 16600642

[34] Andrews-Shigaki BC, Armstrong BSR, Zaitsev M, Ernst T. Prospective Motion Correction for Magnetic Resonance Spectroscopy Using Single Camera Retro-Grate Reflector Optical Tracking. J Magn Reson Imaging. 2011;33(2):498–504. 10.1002/jmri.22467 21274994 PMC3076055

[35] Qin L, van Gelderen P, Derbyshire JA, Jin F, Lee J, Zwart JAd, et al Prospective Head-Movement Correction for High-Resolution MRI Using an in-Bore Optical Tracking System. Magn Reson Med. 2009;62(4):924–934. 10.1002/mrm.22076 19526503 PMC3523280

[36] Maclaren J, Herbst M, Speck O, Zaitsev M. Prospective Motion Correction in Brain Imaging: a Review. Magn Reson Med. 2013;69(3):621–636. 10.1002/mrm.24314 22570274

[37] ArmstrongB SR, Maclaren J, Li Q, Kusik TP, Barrows RT, Andrews-Shigaki B, et al. Distance Estimation by Moving-Window Principal Component Analysis for RGR-Based Subject Motion Tracking in MR Scans. In: Current Concepts of Motion Correction for MRI & MRS. ISMRM Workshop Series 2010; 2010. p. 25 February 2010, 10:20–10:25.

[38] Armstrong BSR. Optical Markers and Detection Accuracy. In: Current Concepts of Motion Correction for MRI & MRS. ISMRM Workshop Series 2010; 2010. p. 26 February 2010, 17:30–18:00.

[39] Maclaren J, Armstrong BSR, Barrows RT, Danishad KA, Ernst T, Foster CL, et al Measurement and Correction of Microscopic Head Motion during Magnetic Resonance Imaging of the Brain. PloS one. 2012;7(11):e48088 10.1371/journal.pone.0048088 23144848 PMC3492340

[40] Clarke TA, Fryer JG. The Development of Camera Calibration Methods and Models. Photogramm Rec. 1998;16(91):51–66. 10.1111/0031-868X.00113

[41] Kadashevich I, Danishad A, Speck O. Automatic Motion Selection in One Step Cross-Calibration for Prospective MR Motion Correction. MAGMA. 2011;24(Supplement 1):266–267.

[42] Herbst M, Lovell-Smith C, Haeublein B, Sostheim R, Maclaren JR, Korvink JG, et al. On the Robustness of Prospective Motion Correction for Clinical Routine. In: Proceedings of the 21st Scientific Meeting: International Society for Magnetic Resonance in Medicine; 2013. p. 3766.

[43] StuchtD, SchulzeP, DanishadKA, KadashevichIY, ZaitsevM, ArmstrongBS, et al. Accuracy of Prospective Motion Correction in MRI Using Tracking Markers on Repositionable Dental Impressions. In: Medical Image Understanding and Analysis 2012: Proceedings of the 16th Conference. Swansea/UK: BMVA; 2012. p. 223–228.

